# Mid-upper arm circumference and body mass index as different screening tools of nutritional and weight status in Polish schoolchildren across socio-political changes

**DOI:** 10.1038/s41598-019-48843-5

**Published:** 2019-08-27

**Authors:** Natalia Nowak-Szczepanska, Aleksandra Gomula, Slawomir Koziel

**Affiliations:** Department of Anthropology, Hirszfeld Institute of Immunology and Experimental Therapy Polish Academy of Sciences, Podwale 75, 50-449 Wroclaw, Poland

**Keywords:** Ecological epidemiology, Socioeconomic scenarios

## Abstract

Intergenerational changes in many biological traits are indicators of environmental conditions. One of such anthropometric measures is the mid-upper arm circumference (MUAC) which estimates nutritional status. Likewise, Body Mass Index (BMI) is widely used as an anthropometric indicator of relative weight. The aim of this study was to reveal secular trends in MUAC and BMI, as biological indicators of changing living conditions, between 1966 and 2012 among Polish children from different socioeconomic groups. Total sample involved 64 393 schoolchildren aged 7–18 years, investigated in 4 Surveys (1966, 1978, 1988, 2012). Overall socioeconomic status (SES) was divided into two categories: lower and higher (including: urbanization, family size, parental education). Results showed that MUAC and BMI differed significantly with respect to the year of survey, sex and SES category. Both measures were higher within higher SES group compared to the lower one until 1988, while in 2012 convergence of these indicators in both SES categories was observed. Both the year of survey, sex, SES category and interactions between them had higher impact on MUAC than BMI (measured by effect size). Our findings revealed that long-term socioeconomic changes affect MUAC more noticeably than BMI. Therefore MUAC may be a more accurate screening tool.

## Introduction

The mid-upper arm circumference (MUAC) is an anthropometric screening tool that is useful especially in monitoring severe undernutrition^1^. It is also a reliable biological indicator of overweight and obesity among children in resource-limited areas^2,3^. Also other authors [e.g.^4^] have shown that MUAC, next to the waist circumference and Body Mass Index (BMI), may be a good indicator of overweight and obesity among children and adolescents. The BMI is a common and universally used measurement for screening both underweight and overweight across many populations [e.g.^5^]. Within a broader context, socioeconomic changes affect values of BMI along with the secular (intergenerational) trend, particularly in countries that underwent political transition [e.g.^6–8^]. In these countries, the economic growth and improvement of living conditions led to, for instance, higher access to food resources and changes in individual’s lifestyle. In the further course, these modifications resulted in excess caloric intake and, finally, higher levels of the overweight and obesity prevalence [see also:^7^]. Moreover, until 1990s in low- and middle income developing countries higher socioeconomic status (measured, for instance, by the educational level) was related to higher values of BMI^9^. After 1990s, in many developed and some developing countries, there was a reversal of this socioeconomic stratification, where lower SES groups revealed higher level of excess weight, mainly due to their easier access to cheap, but high-caloric food products [see: e.g.^9,10^].

After the Second World War, Poland underwent vast economic and socio-political changes, beginning with rising after wartime destruction, through the period of long-lasting communism, political transformation to democracy and free market economy, and, finally, joining the European Union in 2004 [compare:^7,11^]. In the post-war period, Polish economy was under reconstruction, and communist centrally planned economic system was introduced. As a consequence, it has finally brought large disproportions and failures in development of particular economic sectors. Difficult economic conditions during the years of communism were interrupted by temporal improvement due to government’s borrowing of foreign capital at the beginning of 1970s, which, finally, led to massive debts of the country at the end of the 1970s. This economic stagnation resulted in the price increases, food shortages and rationing of goods, where certain items and food products were limited and accessed only by ration cards (introduced by the government in the second half of the 1970s and maintained until the end of 1980s). These adverse living conditions were followed by nationwide strikes, political crisis and opposition from the social movement led by Solidarity union. In the end, a round-table agreement in 1989 established changes in political system. After political transformation towards democracy and free-market economy, between 1990 and 2015 Polish economy doubled in size, in terms of real GDP (gross domestic product), and GDP *per capita* increased from 32% to 60% of the Western European mean value^12^. Abovementioned socioeconomic transitions were associated with changing living standards, including, for instance, differentiated access to food resources, periods of food shortages and, finally, economic as well as individual living improvement after political transformation^7^. As nutritional status of children may accurately reflect environmental changes within the whole population^13^, it is worth identifying a simple biological measure that allows for monitoring the influence of socioeconomic transformations on human growth.

While BMI is a common and widely used measure of monitoring weight status within the population, it is also often criticized for its low accuracy in identifying overweight and obesity among individuals, especially in children and adolescents, because its adequacy varies according to the degree of body fatness and body composition [compare:^14^]. On the other hand, researchers have shown that MUAC can predict overweight and excess fatness with fairly reasonable accuracy^2^. Therefore, the aim of this study was to identify whether MUAC, compared to BMI, may be useful as a biological indicator of nutritional status affected by the socioeconomic temporal changes and differentiated by socioeconomic stratification, based on the cross-sectional data of Polish schoolchildren measured in 1966, 1978, 1988 and 2012. Our research hypothesis is that during analysed period of vast and long-lasting socio-political transitions MUAC has reflected socioeconomic changes and social stratification at least to the same extent as BMI, where, along with the time, there were significant increases in the values of both anthropometric indicators, as well as higher socioeconomic groups revealed higher values of BMI and MUAC than lower ones.

## Material and Methods

### Participants and measurements

This study involved a total sample of 64 393 Polish schoolchildren (32 170 boys; 32 223 girls) aged 7–18 years. Participants were examined in four subsequent cross-sectional Polish Anthropological Surveys in 1966 (N = 19 188), 1978 (N = 19 763), 1988 (N = 19 322) and 2012 (N = 6 120) (note that the number of participants may slightly differ depending on the performed analyses) [see: e.g.^7,8,15^]. Survey locations involved schools located in cities (>100 000 inhabitants), towns (<100 000 inhabitants) and villages (rural areas) in different parts of Poland. The 1966 Survey was based on the nationwide random sample (covering all 98 contemporary districts). Children were selected by the National Bureau of Statistics (GUS) in a multi-stage procedure of sampling in the following order: regions within Poland, the cities, towns and villages within the regions, the schools within those locations, the required number of children within the schools^7,15^. However, because of the high costs of this type of research, next studies involved only three cities (Warsaw, Lodz, Wroclaw) and four towns (Bystrzyca Klodzka, Pinczow, Siemiatycze and Wolsztyn) with the villages located within their county districts: in the cities schools were randomly selected, while in the towns and villages the entire population of schoolchildren was included. Yet, in 2012, in contrast to previous mandatory surveys, a written consent of parents or legal guardians of the children was required, therefore, not entire target population of children has been involved^7^. Finally, data included into this study were derived from the same places of research conducted in 1966, 1978, 1988 and 2012 (although in 1966 study had much wider range). Anthropometric measurements as well as demographic and social characteristics were collected by trained anthropological staff. Height and MUAC were measured to the nearest 0.1 cm, while weight was assessed to the nearest 0.1 kg, using, respectively, an anthropometer, anthropometric tape and weight scale. During these measurements children were examined in light clothes without shoes.

All procedures performed in studies involving children were approved by the *Scientific Ethics Committee at the University School of Physical Education in Wroclaw*, *Poland* and were conducted in accordance with the ethical standards from the Declaration of Helsinki. Informed consent was obtained from parents of all individual participants included in the study (in 2012 in a written form, in previous studies – as an oral consent, since Polish law did not demand written forms earlier).

### Statistical analyses

Descriptive statistics of analysed indices were presented in Table 1 for BMI and Table 2 for MUAC. BMI was calculated as weight (in kilograms) divided by height (in meters squared) and standardized for age (Z-BMI) according to LMS parameters (L for the skewness, M for median value, S for the generalized coefficient of variation), separately for boys and girls^16^. MUAC was also standardized for age (Z-MUAC) using LMS parameters, separately for both sexes^17^. LMS method allows to take into account an effect of age as a covariate that is related to changing distribution of a given measurement during growth of children [for more details see:^18^]. Socioeconomic status (SES) was assessed by the scores of first component derived from the principal component analysis and representing overall SES (eigenvalue of the first factor: 2.47, variance explained by the first factor: 61.68%), including following variables: urbanization level (city, town, village), family size (one, two, three, four or more children), mother’s and father’s education (university, secondary, trade, elementary schools). Overall SES was divided into two categories: higher SES (above median value) and lower SES (below median value). Statistical analyses involved three-way analysis of variance for Z-BMI and Z-MUAC as dependent variables and the year of survey, sex and SES category as independent variables. The interaction effect between the year of survey, sex and SES category was also analysed with respect to its influence on dependent variables. For *post hoc* comparisons between successive Surveys, both sexes and SES categories, Tukey’s HSD test for unequal sample size was implemented (assumed a significance level of *p* < 0.05). Eta squared values (η^2^) were calculated as the measures of effect size of sex, year of survey, SES category and interaction between them on standardized values of BMI and MUAC. All calculations were done using Statistica 13.1^19^.

**Table 1:** Descriptive statistics for Body Mass Index (*N* = number of participants, M = mean value [kg/m^2^], SD = standard deviation) among Polish schoolchildren from Surveys in 1966, 1978, 1988 and 2012.

age	1966				1978				1988				2012			
	*boys*		*girls*		*boys*		*girls*		*boys*		*girls*		*boys*		*girls*	
	*N*	M (SD)	*N*	M (SD)	*N*	M (SD)	*N*	M (SD)	*N*	M (SD)	*N*	M (SD)	*N*	M (SD)	*N*	M (SD)
7	506	15.77 (1.59)	437	15.17 (1.43)	821	15.79 (1.85)	819	15.51 (1.77)	864	15.73 (1.63)	872	15.68 (1.73)	210	16.12 (2.05)	246	16.56 (2.24)
8	645	15.65 (1.50)	663	15.22 (1.59)	881	15.95 (1.79)	791	15.71 (1.86)	909	15.92 (1.78)	866	15.62 (1.77)	346	17.18 (2.69)	336	17.01 (2.58)
9	741	16.00 (1.69)	755	15.41 (1.62)	885	16.28 (2.03)	827	16.20 (2.10)	945	16.38 (1.92)	830	16.20 (2.19)	327	17.85 (2.79)	319	17.39 (2.94)
10	734	16.46 (1.73)	784	15.90 (1.84)	844	16.70 (2.17)	799	16.49 (2.26)	896	16.84 (2.16)	859	16.68 (2.20)	288	18.30 (3.03)	272	17.87 (3.03)
11	856	16.63 (1.72)	868	16.51 (1.88)	828	16.94 (2.22)	823	17.06 (2.45)	814	17.11 (2.31)	816	17.22 (2.52)	309	18.74 (3.27)	326	18.50 (3.03)
12	809	17.13 (1.85)	938	17.07 (2.05)	847	17.51 (2.41)	786	17.76 (2.41)	834	17.58 (2.51)	865	17.96 (2.69)	264	19.20 (3.46)	237	19.10 (3.24)
13	886	17.69 (1.93)	877	17.80 (2.20)	842	18.17 (2.57)	809	18.57 (2.56)	799	18.34 (2.73)	774	18.53 (2.64)	198	20.06 (3.44)	217	20.09 (3.46)
14	819	18.27 (1.89)	837	18.98 (2.32)	816	18.70 (2.32)	826	19.76 (2.64)	736	18.82 (2.61)	771	19.77 (2.93)	167	20.53 (3.66)	200	19.89 (2.86)
15	1143	19.40 (2.21)	1040	20.09 (2.38)	854	19.56 (2.50)	875	20.27 (2.48)	959	19.85 (2.49)	855	20.43 (2.72)	147	21.20 (3.40)	157	20.46 (2.53)
16	1096	20.10 (2.18)	1101	20.76 (2.32)	836	20.37 (2.36)	906	20.68 (2.44)	893	20.77 (2.38)	796	21.22 (2.73)	240	22.18 (3.62)	303	21.13 (3.14)
17	873	20.84 (2.03)	969	21.21 (2.33)	764	21.05 (2.32)	862	21.12 (2.30)	727	21.42 (2.51)	722	21.60 (2.67)	281	22.56 (3.32)	304	21.30 (3.25)
18	397	21.14 (2.13)	414	21.46 (2.27)	650	21.34 (2.05)	772	21.27 (2.22)	449	21.84 (2.29)	471	21.56 (2.50)	195	23.05 (3.36)	231	21.40 (3.24)
total *N*	9505		9683		9868		9895		9825		9497		2972		3148

**Table 2:** Descriptive statistics for mid-upper arm circumference (*N* = number of participants, M = mean value [cm], SD = standard deviation) among Polish schoolchildren from Surveys in 1966, 1978, 1988 and 2012.

age	1966				1978				1988				2012			
	*boys*		*girls*		*boys*		*girls*		*boys*		*girls*		*boys*		*girls*	
	*N*	M (SD)	*N*	M (SD)	*N*	M (SD)	*N*	M (SD)	*N*	M (SD)	*N*	M (SD)	*N*	M (SD)	*N*	M (SD)
7	506	16.92 (1.32)	437	17.26 (1.55)	821	18.26 (1.91)	819	17.99 (1.77)	864	17.80 (1.70)	872	17.97 (1.78)	210	18.34 (2.13)	246	18.99 (2.10)
8	645	17.26 (1.48)	663	17.87 (1.82)	881	18.75 (1.87)	791	18.49 (1.89)	909	18.36 (1.85)	866	18.18 (1.83)	346	19.60 (2.61)	336	19.78 (2.46)
9	741	17.86 (1.68)	755	18.41 (1.83)	885	19.26 (2.06)	827	19.33 (2.20)	945	19.02 (2.02)	830	19.06 (2.19)	327	20.60 (2.71)	319	20.40 (2.92)
10	734	18.54 (1.81)	784	19.13 (2.02)	844	20.08 (2.27)	799	19.86 (2.19)	896	19.81 (2.20)	859	19.83 (2.29)	288	21.50 (3.06)	272	21.38 (2.83)
11	856	18.99 (1.84)	868	19.94 (2.08)	828	20.57 (2.32)	823	20.66 (2.35)	814	20.34 (2.30)	816	20.68 (2.47)	309	21.96 (3.06)	326	22.14 (2.97)
12	809	19.71 (1.95)	938	20.71 (2.31)	847	21.37 (2.58)	786	21.55 (2.36)	834	21.19 (2.49)	865	21.46 (2.46)	264	22.93 (3.44)	237	22.87 (2.82)
13	886	20.49 (2.03)	877	21.66 (2.50)	842	22.26 (2.66)	809	22.30 (2.52)	799	22.24 (2.65)	774	22.06 (2.51)	198	24.13 (3.44)	217	24.10 (3.05)
14	819	21.47 (2.16)	837	22.90 (2.65)	816	23.25 (2.52)	826	23.59 (2.46)	736	23.05 (2.68)	771	23.27 (2.72)	167	24.79 (3.43)	200	23.84 (2.86)
15	1143	22.99 (2.45)	1040	24.22 (2.73)	854	24.44 (2.58)	875	24.07 (2.27)	959	24.39 (2.60)	855	23.94 (2.58)	147	26.03 (3.07)	157	24.67 (2.70)
16	1096	24.14 (2.37)	1101	24.89 (2.56)	836	25.60 (2.53)	906	24.60 (2.29)	893	25.51 (2.44)	796	24.46 (2.36)	240	26.99 (3.12)	303	25.09 (2.83)
17	873	25.19 (2.22)	969	25.35 (2.57)	764	26.48 (2.29)	862	24.96 (2.12)	727	26.52 (2.67)	722	24.73 (2.35)	281	27.60 (3.06)	304	25.22 (2.84)
18	397	25.66 (2.25)	414	25.60 (2.45)	650	27.02 (2.16)	772	25.17 (2.13)	449	26.99 (2.23)	471	24.83 (2.30)	195	28.43 (2.93)	231	25.31 (3.06)
total *N*	9505		9683		9868		9895		9825		9497		2972		3148	

## Results

**Figure 1:**
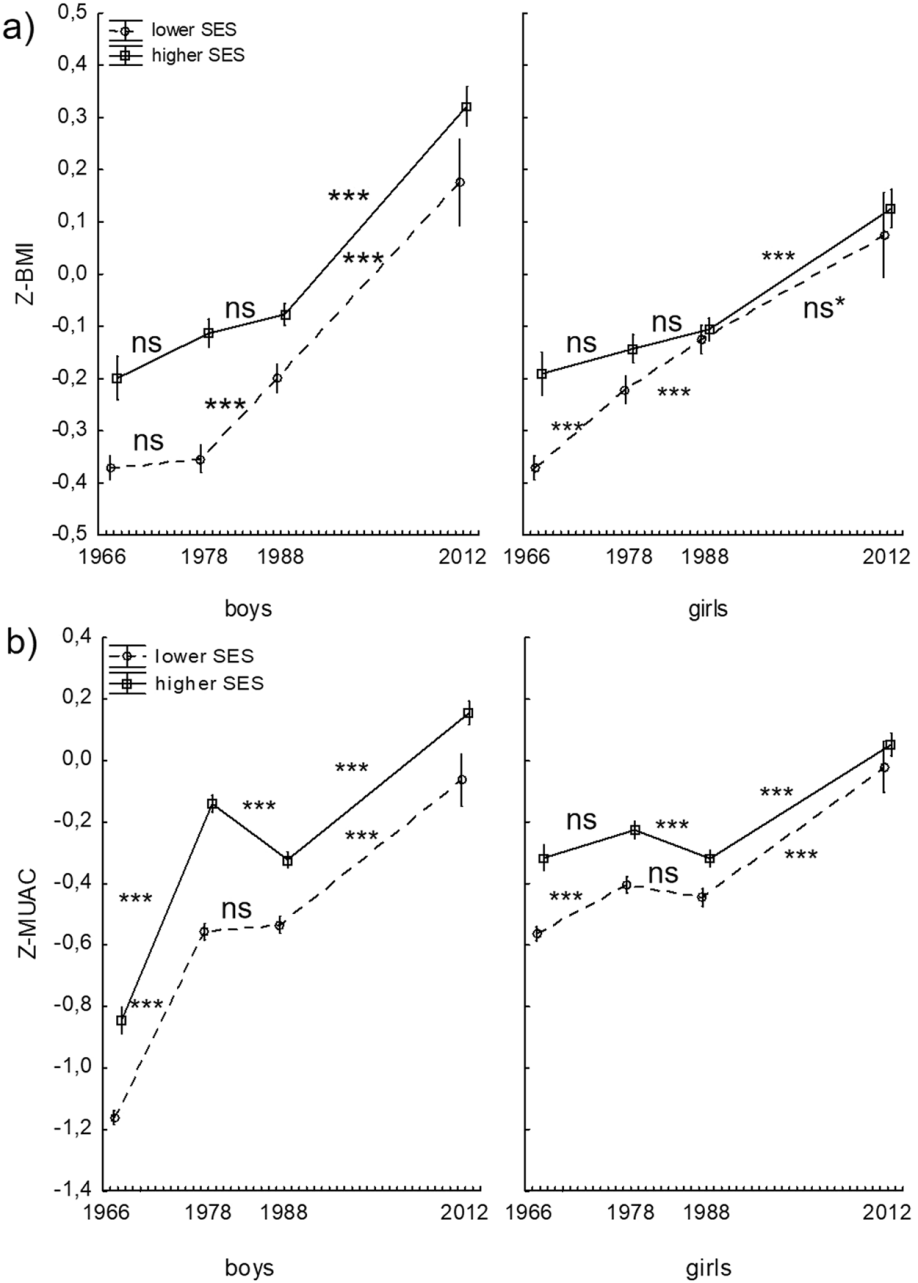
(**a**) Body Mass Index standardized for age (Z-BMI) and (**b**) mid-upper arm circumference standardized for age (Z-MUAC) in boys and girls from two different SES categories between 1966 and 2012 (vertical lines indicate 95% CI for the mean values; ***p < 0.001, **p < 0.01, *p < 0.05, ns*p = 0.08, ns: non-significant; please note that reported *p-*values refer here only to the *post-hoc* differences between subsequent Surveys).

Both the year of survey, sex, SES category and the interactions between them had a significant effect on Z-BMI (F(3, 63608) = 5.17, p < 0.01; except for sex, where p = 0.08; Fig. 1a) and Z-MUAC (F(3, 58918) = 6.94, p < 0.001; Fig. 1b). For Z-BMI in both SES categories for boys and girls between 1966 and 2012 rather constant and significant increase (p < 0.001; especially between 1988 and 2012) or no significant changes (for higher SES category in both sexes during the years 1966–1978 and 1978–1988 as well as for lower SES category in boys between 1966 and 1978) in its value were observed. Meanwhile, during the years 1966–1978 and 1988–2012, Z-MUAC significantly increased in both sexes (p < 0.001, except for higher SES category in girls between 1966 and 1978, where no significant change was observed), but between 1978 and 1988 a significant decrease in its value (for higher SES category; p < 0.001) or no significant changes (for lower SES category; p > 0.05) were noted.

The *post hoc* comparisons rarely revealed significant sex differences for Z-BMI (lower BMI was observed in boys only in a lower SES category in 1978 and 1988, while in 2012 higher BMI in boys was noted in a higher SES category; p < 0.05). On the other hand, for Z-MUAC almost all differences between boys and girls in each Survey were significant (p < 0.05). Z-MUAC was higher in girls in 1966 in both SES categories as well as in 1978 and in 1988 in lower SES category (p < 0.01), but in 2012 its value significantly increased in favour of boys from higher SES category (p < 0.05).

Socioeconomic differences for both Z-BMI and Z-MUAC in boys and girls remained constant between 1966 and 1988 in favour of higher SES category (p < 0.01; except for Z-BMI in girls in 1988, where no significant SES differences were noted). In 2012 differences between SES categories for both anthropometric indices diminished and became non-significant (p > 0.05), except for Z-MUAC in boys still in favour of higher SES group (p < 0.05). Eta squared values (*η*^2^) were much higher for Z-MUAC than for Z-BMI for all analysed independent variables (for sex: 0.0036 *vs* 0.0001, for the year of survey: 0.0348 *vs* 0.0109, for SES categories: 0.0076 *vs* 0.0024; for the interaction effect of all analysed variables: 0.0004 *vs* 0.0002, respectively).

## Discussion

In this study, a significant effect of the year of survey, sex, SES category and the third-order interaction between them on both Z-BMI and Z-MUAC has been noted (p < 0.01 and p < 0.001, respectively). However, between 1978 and 1988 Z-BMI significantly increased in boys and girls from lower SES category (p < 0.001) or did not change in those from higher SES groups, while Z-MUAC significantly decreased during this period in children of both sexes from higher SES category (p < 0.001) or did not change significantly among those from lower SES groups. Moreover, significant sex differences were observed more frequently for Z-MUAC than for Z-BMI. With respect to the SES category, between 1966 and 1988 constant social gradient was observed for both Z-BMI and Z-MUAC in favour of higher SES groups. In 2012, SES differences for analysed anthropometric indicators mainly diminished and lost their significance. Our results also showed that all analysed independent factors revealed higher effect size on Z-MUAC than on Z-BMI.

Z-BMI was mostly constantly increasing throughout the studied period (1966–2012), while Z-MUAC had a significant decrease in its value between 1978 and 1988. These findings may indicate that MUAC, in comparison with BMI, is a more sensitive anthropometric indicator of long-term socioeconomic changes that may affect human growth. Analogously, in the study describing secular changes of BMI in particular age categories in children from 7 to 18 years of age, Gomula *et al*.^7^ found some significant increases in BMI between 1978 and 1988, but they were observed in a few age classes in boys and girls. However, no significant decrease was noted. From a historical point of view, the period between Surveys in 1978 and 1988 was particularly difficult for a Polish society. During the communism, at the turn of the 1970s and 1980s, there was an economic crisis in Poland with a decrease in a real income of households, food shortages and price increases. In 1976 first ration cards were introduced to distribute limited food products and other goods. Our study has shown that these difficult living conditions were unfavourable for nutritional status of children, but affected more significantly the mean values of arm circumference than relative weight. Moreover, in the period after the Second World War, a difference in Z-MUAC in 1966 between boys and girls was distinctly visible in favour of girls (see: Fig. 1b), as males in the post-war years, due to their higher level of biological eco-sensitivity, might be more vulnerable to the difficult conditions during the rebuilding of the country. During the same time, there was no significant difference in Z-BMI between the sexes. Therefore, MUAC seems to be a more reliable indicator of historical socioeconomic changes than BMI. Moreover, researchers concerning monitoring severe acute malnutrition in African and Asian populations have shown that MUAC itself may be also more sensitive (at high specificity levels) anthropometric measure than weight-for-height z-score for identifying children at high risk of death^1^. These results support our research findings confirming that body weight might be a not ideal measure in population studies.

Although Z-MUAC seems to reflect long-term socioeconomic changes more accurately than Z-BMI, the differences between these two indicators with respect to the current higher or lower SES categories are not so pronounced. Both Z-MUAC and Z-BMI revealed constantly higher values in higher SES group compared to the lower SES category between 1966 and 1988 (except for Z-BMI in girls from 1988, where no significant differences were noted; see: Fig. 1a,b). This phenomenon of positive correlation between nutritional or weight status and socioeconomic level was also observed in other low- and middle income developing countries until 1990s^9^. Nevertheless, in 2012 socioeconomic differences diminished for both anthropometric indicators, except for Z-MUAC in boys, where it was slightly, but still significantly, higher in higher SES group. However, the convergence or even reversed social stratification of biological measures were recently observed also in other, particularly developed, countries, where higher socioeconomic status, assessed by the higher urbanization level or higher parental education, became a protective factor against excess weight [e.g.^20,21^].

Some researchers have shown that MUAC is strongly correlated with adiposity indicators in both sexes^3^, while BMI in childhood has only a moderate association with adiposity index [but still significant; see:^22^]. Our results seem to be in accordance with these findings, since significantly higher values of Z-MUAC among girls during the years 1966–1988 might reflect higher content of adipose tissue in females, while BMI mostly did not differ significantly with respect to sex during corresponding period. Otherwise, higher values of both Z-MUAC and Z-BMI in favour of boys from higher SES category in 2012 might be related to a general higher increase of excess fatness among boys after the political transformation in Poland^7^. Between the years 1988 and 2012 an increase in excess weight was noted in boys in all groups of age, while in adolescent girls no significant changes in BMI were observed. As Gomula *et al*.^7^ concluded, after the transition adolescent girls became thinner because of social and cultural pressure towards pattern of beauty promoting slimness. On the other hand, after socio-political transformation, higher values of both anthropometric indicators in boys compared to girls (only from higher SES category) and their persistent social stratification with respect to Z-MUAC may suggest that these boys were more sensitive to the influence of economic welfare, that mainly affected the most privileged groups.

Our study had a limitation related to a decreased number of participants in the Survey conducted in 2012 compared to previous Surveys. It was due to the fact that parents were less willing to provide written consent in studies on their children in 2012. One of the reasons might be that they were afraid of the effect of stigmatization associated with the assessment of body weight in their children [for more details see:^7^], although it was noted anonymously. However, this limitation was taken into account statistically by using *post-hoc* comparisons for unequal sample sizes between Surveys (see: *Statistical analyses*). Another limitation involved a long time interval between studies in 1988 and 2012, because there was a lack of data collection among schoolchildren from the 1990s, shortly after the onset of political transformation in Poland. Therefore, it is difficult to define, whether the secular increases in body weight and arm circumference were observed continuously from 1988 until 2012, or these measures increased, for instance, in 1990s and then stabilized. Nevertheless, other studies on Polish schoolchildren (from Cracow) have shown that their relative body weight was gradually increasing from 1970s until the last years^23^, hence, this continuous increase in anthropometric measurements could have probably been similar in our studied population between 1990s and 2012.

Increasing prevalence of overweight and obesity among children is an important public health issue, because it leads to a greater risk of obesity-related diseases in both youth and adult life [e.g.^24,25^]. Detrimental effects of underweight are also well known for growth and development during childhood, since it may cause nutritional deficits and lead to the impairment of immune system with more frequent infections [e.g.^26^]. Therefore, it is important to find a simple and reliable anthropometric measure, other than BMI, that indicates both over- and underweight. Particularly, monitoring nutritional status among children should be one of the most important priorities of public health institutions. Since MUAC, compared to BMI, reflects more accurately long-term socioeconomic changes affecting growth of children, it may be a more reliable and sensitive anthropometric indicator in population screening for epidemiological purposes. Therefore, MUAC, as a screening tool, may be useful in predicting not only severe malnutrition and, on the other hand, overweight and obesity, but also may reflect long-lasting socioeconomic and cultural changes within a whole population. It is a very simple and non-invasive measure^27^ that could be reasonably informative. Since MUAC closely reflects body fat tissue ^28^, it can be the most practical index in epidemiological studies measured in almost every situation, providing the information about the risk of metabolic and cardiovascular diseases in overweight and obese individuals. It is worth to emphasize that differences between MUAC and BMI due to their different sensitivity to socioeconomic conditions are more visible during long-term historical changes, while the current effect of socioeconomic stratification seems to be analogous for both anthropometric indicators (although it is slightly more pronounced in Z-MUAC among boys). Nevertheless, MUAC may successfully allow for rapid collection of nutritional data in every setting and facilitate population screening for both under- and overweight with respect to the individual’s socioeconomic conditions and their changes.

## Data Availability

The dataset analysed during the current study is available from the corresponding author on reasonable request.
